# Molecular Imaging: A Promising Tool to Monitor Islet Transplantation

**DOI:** 10.1155/2011/202915

**Published:** 2011-10-15

**Authors:** Ping Wang, Zdravka Medarova, Anna Moore

**Affiliations:** Molecular Imaging Laboratory, MGH/MIT/HMS Athinoula A. Martinos Center for Biomedical Imaging, Massachusetts General Hospital and Harvard Medical School, Charlestown, MA, 02129, USA

## Abstract

Replacement of insulin production by pancreatic islet transplantation has great potential as a therapy for type 1 diabetes mellitus. At present, the lack of an effective approach to islet grafts assessment limits the success of this treatment. The development of molecular imaging techniques has the potential to fulfill the goal of real-time noninvasive monitoring of the functional status and viability of the islet grafts. We review the application of a variety of imaging modalities for detecting endogenous and transplanted beta-cell mass. The review also explores the various molecular imaging strategies for assessing islet delivery, the metabolic effects on the islet grafts as well as detection of immunorejection. Here, we highlight the use of combined imaging and therapeutic interventions in islet transplantation and the in vivo monitoring of stem cells differentiation into insulin-producing cells.

## 1. Introduction

Type 1 diabetes mellitus (T1DM) is characterized by an absolute deficiency of insulin secretion with hyperglycemia as a consequence. T1DM is one of the most common diseases of childhood. 13,000 new cases are diagnosed each year in North America [1]. The first major breakthrough in the treatment of T1DM was the isolation of insulin and use of its synthetic forms. Although insulin has changed the clinical course of TIDM from an acutely fatal disease to a chronic one with severe long-term complications, it does not cure diabetes [2]. In 1966, the first whole pancreas transplantation was performed [3]. Clinical studies show that pancreatic allotransplantation offers superior glycemic control for T1DM and prevents or even reverses secondary complications, including nephropathy [4]. The elevated risk of surgical complications and the relative invasiveness of the procedure, however, makes the practice of solid organ transplantation rare in T1DM patients. Since 1999, when the first accounts of consistent success in restoring normoglycemia using islet transplantation appeared, this less invasive procedure has become an important alternative treatment for T1DM patients.

The first attempt to transplant an islet cell xenograft was performed in 1893, 29 years before the isolation of insulin. A 15-year-old diabetic patient was transplanted sheep pancreatic tissue beneath his skin. The procedure failed and the patient died after 3 days [5]. In 1967, a method of isolating islets using collagenase was described [6]. This launched the earliest islet transplantation in animal models in 1972 [7]. The first clinical islet allograft was performed in 1974 [8]. The next 25 years have witnessed attempts to achieve normoglycemia in type 1 diabetic patients using islet transplantation, with limited success. In 1999, the Edmonton protocol revived interest in this procedure by reporting reproducible success in terms of insulin independence through islet transplantation [9]. All of the patients maintained insulin independence after 1 year of followup [10]. This new protocol relies on a prednisone-free immunosuppressive regimen and improved islet delivery through intraportal infusion of freshly isolated islets, followed by a second or third infusion of additional islets [8]. With this protocol, from 1999 to 2008, almost 400 patients received allogeneic islet transplants [11]. However, with more follow-ups, it became apparent that insulin independence was only transient in most recipients. A recent international trial using the Edmonton protocol showed that only 44% of the patients receiving islet transplantation remained insulin independent at one year after-transplant [12]. Unfortunately, only less than 10% of the recipients remain insulin independent for up to 5 years [13].

It is believed that many factors contribute to the loss of graft function. Early losses are primarily linked to damage sustained during the isolation procedure or in the graft microenvironments, secondary to ischemia-reperfusion-like injury and nonspecific inflammation such as the instant blood-mediated inflammatory reaction (IBMIR) [14]. Subsequent losses are usually more progressive and involve several immunological related factors. The presence of allogeneic rejection has been strongly suggested by the poorer clinical outcomes in case of prior human leukocyte antigen sensitization and, conversely, by favorable results achieved with more potent immunosuppression treatment [15]. The recurrence of autoimmunity also is a major limiting factor in long-term survival of islet grafts. Some studies have shown that monocytic infiltration of the graft occurs as early as 14 days after transplantation, with a preferential loss of insulin-secreting beta cells [16]. After islet transplantation, elevated islet cell autoantibody titers (to glutamic acid decarboxylase or GAD65) persisted [17]. Immuosuppressive drug toxicity is another immune-related assault on the graft. It has been demonstrated that rapamycin, at a concentration usually used to prevent islet grafts rejection, is able to reduce the rate of beta cell proliferation not only in transplanted rat islets but also in host murine islets, suggesting that the progressive islet grafts dysfunction observed under immunosuppressive therapy may result in part from an impairment in beta cells regeneration [18]. 

It is clear by now that an effective approach to islet grafts assessment following transplantation is urgently needed. Successful monitoring of the graft would allow us to test the viability and functionality of the graft. Such monitoring would also provide a better understanding of the various mechanisms involved in graft loss. It would also permit us to design and implement prompt intervention and more carefully tailored treatment. However, currently the majority of methods for assessment of the islet grafts are still indirect. These include indicators of metabolic control, such as fasting and stimulated glucose levels, oral glucose tolerance testing (OGTT), C-peptide levels, HbA1c levels, mean amplitude glycemic excursions (MAGE), and insulin secretion. In addition, immune events and complications associated with islet transplantation are indirectly tested as well. These include allo- and autoimmune antibodies as well as signs of toxicity or impairment of liver function [19]. All of these indirect parameters only provide information on the late stages of graft rejection. Since the mechanisms behind islet function represent a finelytuned network of regulated interactions and feedback loops, alterations in C-peptide and insulin release do not become apparent until most islets have already been destroyed [20–22]. The only direct morphological assessment of transplant fate in the clinic is obtained through histological biopsy. However, it could not be widely applied due to the small size of islet grafts and their relatively low frequency dispersed in a large organ such as the liver. Moreover, this approach is invasive. Therefore, it is critical to establish a noninvasive method to monitor the fate of islets directly in a clinical setting. 

Molecular imaging is a rapidly emerging biomedical research discipline. Considerable efforts have been directed in recent years toward the development of noninvasive high-resolution in vivo imaging technologies including optical imaging, nuclear imaging, and magnetic resonance imaging (MRI). At the same time, various molecular imaging probes with greater specificity and targeting potential have been designed and tested (antibodies, ligands, or substrates that can specifically interact with targets in particular cells or subcellular compartments) [23]. The development of molecular imaging techniques has the potential to fulfill the goal of real-time non-invasive monitoring of the functional status and viability of the islet grafts after transplantation. The present paper explores the various preclinical and clinical molecular imaging strategies for the tracking of graft fate, islet delivery strategies, as well as detection of immunorejection. We also review the use of combined imaging and therapeutic approaches in islet transplantation [24] and the in vivo monitoring of embryonic stem cells differentiation into insulin-producing cells.

## 2. Imaging Beta Cells as a Key Step towards Understanding Islet Transplantation

Progress towards the goal of direct assessment of graft integrity, viability, and function would rest on previous experience in the area of pancreatic islet imaging as a tool for measurement of islet mass and integrity. Imaging beta cells represents a daunting challenge both from a biological and a technological perspective, owing to the innately complex structure and distribution of pancreatic islets within the pancreas and the elaborate dynamic nature of their metabolic function. Pancreatic islets are small structures (100–400 *μ*m in diameter) that are dispersed throughout the pancreas and constitute only 2% of the pancreatic volume [25]. A variety of imaging modalities have shown promise exclusively in animal models. 

### 2.1. Bioluminescence and Fluorescence Optical Imaging

In 2003, Hara et al. [26] generated transgenic mice that express green fluorescent protein (GFP) under the control of the mouse insulin I gene promoter (MIP). Histological studies showed that the MIP-GFP mice had normal islet architecture with coexpression of insulin and GFP in islet beta cells. GFP-expressing beta cells could be then imaged in vivo allowing for beta cell mass determination. Our group described the ex vivo imaging of beta-cell apoptosis with a near-infrared (NIR) probe (Cy5.5-labeled annexin V) in 2005 [27]. Following, we described the synthesis and testing of an NIR probe for imaging beta-cells in pancreatic islets, which was based on the beta-cells-specific ligand streptozotocin (STZ) labeled with Cy5.5. We observed a bright fluorescence signal consistent with intracellular accumulation of the probe, which was mediated by the glucose transporter 2 (GLUT2 transporter) [28]. Another recent example of a fluorescent probe for detecting beta cells is a near-infrared fluorescent exendin-4 analogue with specificity for the Glucagon-like peptide 1 (GLP-1) receptor on beta cells. Following intravenous administration into mice, pancreatic islets were readily distinguishable from exocrine pancreas, achieving target-to-background ratios within the pancreas of 6 : 1 using intravital microscopy [29]. Bioluminescence imaging of beta-cell mass was applied by Virostko et al. [30] in a recent study where a transgenic mouse model expressing luciferase under control of the mouse insulin I promoter (mouse insulin promoter-luciferase-Vanderbilt University (MIP-Luc-VU)) was used. This model enabled non-invasive assessment of changes in beta-cell mass after islet transplantation based on changes in bioluminescence signal.

### 2.2. Nuclear Imaging

An attractive approach to image beta cell is nuclear imaging. The major advantage of nuclear imaging is its high sensitivity. It relies on radionuclide-labeled contrast agents that target beta cells based on cell-specific antigens, receptors, metabolites, or pharmacologic agents. 

Nuclear imaging has been utilized by our group to estimate beta-cell mass using an ^111^In-labeled monoclonal antibody targeting the beta-cell surface antigen IC2 [31]. In this work ^111^In-labeled IC2 was characterized in vitro in islets as well as in vivo in a diabetic mouse model. Other examples of agents that target beta cells directly through surface antigens include ^125^I-labelled monoclonal antibody R2D6 directed against gangliosides on the plasma membranes of pancreatic beta cells [32] as well as phage-display-derived peptides [33]. In order to overcome some of the weaknesses of monoclonal antibodies as radiotracers, single-chain antibodies (SCAs) were developed. It was reported that removal of the Fc portion to produce an antibody fragment reduced the nonspecific binding to beta cells [34, 35]. 

 Extensive nuclear imaging studies have been performed targeting receptors expressed on beta-cell surface. The vesicular monoamine transporter 2 (VMAT2) is a monoamine transporting integral membrane protein expressed by rodent and human beta cells [36]. Tetrabenazine (TBZ) and Dihydrotetrabenazine (DTBZ) specifically bind to the synaptic VMAT2 [37]. A clinical study showed a reduction in pancreatic uptake of ^11^C-DTBZ in long-standing T1D patients compared to the uptake in the pancreas of healthy control subjects [38]. DTBZ compounds labeled with ^18^F were developed to overcome the short half-life of ^11^C-labeled DTBZ (*T*1/2 = 20 min). Preclinical studies showed high pancreatic uptake of ^18^F-DTBZ in healthy rats with favorable biodistribution leading to improved target-to-background ratios [39]. Recently another new DTBZ derivative, 18F-FP-(+)-DTBZ, was tested. The result showed that this compound had higher pancreatic uptake and lower uptake in the nontarget tissues, especially the liver [40]. The glucagon-like peptide-1 (GLP-1) receptor is a potential target for beta cells imaging as well. It is triggered after binding of the agonists Exendin-3 and Exendin-4 [41]. The first preclinical study using ^123^I-labeled Exendin showed high uptake in the pancreas and in subcutaneous insulinomas [42]. Furthermore, it has been shown that the uptake of ^111^In-DTPA-Lys40-Exendin-3 correlates with beta-cell mass in a linear manner in diabetic rats and that the pancreas can be visualized by SPECT imaging on a dedicated microSPECT scanner [43, 44]. Radiolabeled pharmacologic agents targeting other receptors also have been investigated. They include ligands to sulfonylurea receptors, such as glyburide or tolbutamide analogs [45–47]. 18F-L-DOPA [48] targeting dopamine receptor may also be a suitable approach for the detection of beta-cells.

Various tracers based on radiolabeled glucose have also been explored for targeting beta cells and, of those, the most promising has proven to be mannoheptulose and tritiated D-mannoheptulose, which is apparently transported into cells mainly at the intervention of GLUT-2 [49–51].

### 2.3. MR Imaging

One of the most logical modalities to explore for imaging of pancreatic islets is MRI. MRI does not utilize ionizing radiation, has tomographic capabilities, can deliver the highest-resolution images in vivo, and has unlimited depth penetration. MR imaging has an overall low sensitivity in detecting molecular probes (10^−3^–10^−5^ M) [23]. However, this drawback can be overcome by the application of contrast agents that amplify the signal. New agents such as a novel class of lanthanide complexes for labeling beta cells were first reported at the 2003 NIH Workshop on Imaging Pancreatic Beta cells [52]. ^1^H NMR spectroscopy has been suggested for measuring choline levels, which can possibly indicate the number of viable cells in islet grafts [53]. One group's studies demonstrated that in vitro ^1^H NMR imaging could be used to visualize islets or *β*TC3 cells within their encapsulated environment. They also showed that localizing implanted microencapsulation-based bioartificial pancreas in vivo was feasible with the use of diffusion-weighted imaging [54]. C13 spectroscopy has also been applied for studying glucose-stimulated insulin secretion and has shown promise for the investigation of beta-cell function [55]. Another interesting approach demonstrated the feasibility of direct imaging of beta-cell activation in the presence of divalent manganese cations Mn^2+^ [56, 57]. Recently, Mn^2+^-enhnced MRI was applied for noninvasive detection of beta cell function after glucose infusion. Serial inversion recovery MRI was subsequently performed to probe for Mn^2+^ accumulation in pancreas. This experiment demonstrated the potential of Mn^2+^-enhanced MRI for noninvasive monitoring of beta cell function [58].

A multimodal approach for imaging beta cells was applied by Yong et al. [59]. Transgenic MIP-TF C57/BL6 mice were generated in which beta-cells express a fusion of three different imaging reporters. Multimodal imaging of MIP-TF pancreatic beta-cells was demonstrated by fluorescence microscopy, BLI, and microPET. The MIP-TF mice enabled noninvasive monitoring of beta-cells in models of type 1 and type 2 diabetes. This multimodality imaging animal model might expedite studies in a broad range of diabetes research.

Despite these encouraging examples, however, in the authors' opinion, the issue of visualizing beta-cell mass in vivo using noninvasive imaging remains an unsolved challenge. The ultimate solution to this problem would likely require the identification of specific beta-cell markers and targeting ligands, as well as improvements in imaging technology to permit the acquisition of quantitative information with both high sensitivity and high spatial resolution. The latter would probably rely on the development of multimodality approaches and would demand sophisticated image analysis tools to monitor even small changes in the signal reflective of the dynamic nature of beta-cell mass.

## 3. Imaging of Transplanted Islets—Current Progress

The possibility of directly imaging transplanted islets rests on the fact that isolated islets can be labeled before-transplant using various approaches, including genetic modification with fluorescent or bioluminescent reporters, labeling with exogenous contrast agents, such as superparamagnetic iron oxides for MRI or radiolabeled metabolites for nuclear imaging. These general strategies for the monitoring of transplanted islets by noninvasive imaging have proven valuable in answering questions about graft fate, designing new therapeutic interventions, and testing alternative transplant sites, all ultimately aimed at extending graft longevity and function. 

Here, we highlight some of the progress made using different imaging modalities in acquiring new knowledge about islet transplantation. These studies represent just the first steps towards realizing the true potential of noninvasive imaging to tackle biological questions. The unique advantage of noninvasive imaging lies in its capacity to provide information in authentic physiologic environments, in real-time, and on a systemic level.

### 3.1. Bioluminescence and Fluorescence Optical Imaging

The first evidence of the feasibility of imaging transplanted islets noninvasively was obtained using bioluminescence imaging (BLI). Several laboratories [60–62] reported proof-of-principle studies in which isolated rodent or human islets were genetically engineered to express luciferase and imaged following transplantation. In islets transplanted underneath the renal capsule of immunocompromised mice, the magnitude of the signal was dependent on the islet dose, indicating that the collected information could be used to obtain accurate quantitative information about islet number over time. Although adenovirus-directed luciferase expression attenuated, consistent with the transient nature of the vector, lentivirus vectors could be used to direct the long-term expression of reporter genes in transduced islets. Furthermore, the functionality of transduced islets was retained since transplanted lentivirus-transduced islets led to long term restoration of euglycemia in diabetic mice. Finally, these studies provided new information about islet fate following transplantation. Luciferase signal emanating from the graft remained stable for at least 140 days, indicating graft stability in this model of transplantation [62]. The feasibility of monitoring transplanted islets by BLI was also demonstrated in the intrahepatic transplantation model. Long-term monitoring of adenovirus-transduced islets transplanted into the livers of immunocompromised mice, suggested that, in the described animal model, the intrahepatic islet grafts were also stable, [63]. Early detection of graft rejection was also possible using BLI [64, 65]. 

The first study of the fluorescence optical imaging of islet grafts was published in 2003 [66]. In this study, the graft was monitored with a fused fluorescent reporter protein. In 2006, our group reported that islets incubated with nanoparticle probes (labeled with near-infrared fluorescent Cy5.5 dye) could be detected in the near-infrared channel under the kidney capsule in vivo after transplantation [67].

Despite the lack of a clinical equivalent of these modalities, the information gained from BLI and fluorescence imaging represents a variable contribution to the field of islet transplantation. Therefore, these studies demonstrated the feasibility of conducting preclinical research in small animals in order to address some basic questions about transplanted islet biology and begin to develop alternative strategies for the enhancement of graft function.

### 3.2. Nuclear Imaging

Unlike bioluminescence imaging, nuclear imaging has a clinical equivalent and therefore is more likely to evolve into a modality routinely used in hospitals for monitoring of patients that receive islet transplantation therapy. Initial reports regarding the application of radionuclide imaging to monitor transplanted pancreatic islet grafts employed islets genetically engineered to express a mutant herpes simplex virus type 1 thymidine kinase (sr39tk). The expressed enzyme can phosphorylate positron-emitting, radionuclide-labeled thymidine analogues or acycloguanosine substrates once they are delivered inside the cell, leading to trapping of the phosphorylated product and its detection by positron emission tomography. In one of the early studies, islets were transduced with an Adeno-Tkm adenovirus engineered to express sr39tk under a constitutive promoter [69]. Mice were subjected to microPET imaging after injection of the sr39tk substrate [^18^F] FHBG. As observed by BLI [62], the signal was unstable after a few weeks, likely due to the transient expression of adenovirally directed reporter genes. By contrast, lentiviral transduction of pancreatic islets with sr39tk was used for the long-term monitoring of transplanted islet fate [70]. Islets implanted in the liver were detectable for several weeks after transplantation, suggesting the persistence of the graft. A similar approach was used to monitor the expression of a therapeutic gene (interleukin-10), which could prolong the survival of islet grafts under the kidney capsule of diabetic mice [71]. Although very valuable from a research perspective the reporter nuclear imaging studies are not likely to apply in a clinical scenario in the near future since they involve genetic modification of the islets before-transplant. 

A more clinically relevant technique for the PET imaging of early posttransplant events involved labeling of rodent islets with 2-[^18^F] fluoro-2deoxy-D-glucose (FDG) and their subsequent implantation in the livers of syngeneic rats. The grafts were detected for up to 6 hours [72]. This model, however, is only useful for the short-term monitoring of transplanted islet fate, since the persistence of the label in the islet cells is unknown. Furthermore, the short half-life of ^18^F (110 min) adds to the difficulty of long-term graft monitoring. Without a clear understanding of how long the islets retain the label if at all, it is difficult to obtain quantitative information about islet abundance through time, using this method. In subsequent studies performed in large animals, only *∼*50% of the administered radioactivity was observed in the liver at the end of islet infusion, likely due to islet damage or FDG leakage from the islets [73]. Nevertheless, the clinical feasibility of detecting transplanted islets labeled with [^18^F] FDG was demonstrated for the first time in 2007 [74]. The same group reported that islets labeled with [^18^F] FDG transplanted to 6 patients could be detected during the first 1-2 h. Beyond this, the radioactive half-life and retention within the islets limit the use of this method [68]. Recently, clinical testing of GLP-1 receptor was applied for imaging of human beta cells transplanted in patient muscle, which showed its potential to assess islet survival in clinical transplantation [44]. However, long-term monitoring after-transplant presents a significant problem associated with this method.

### 3.3. Magnetic Resonance Imaging

Magnetic resonance imaging (MRI) is a modality, which, like nuclear imaging, has a broad clinical applicability and can be used to monitor transplanted pancreatic islets. Whereas nuclear imaging is characterized by high sensitivity to contrast agent abundance and a highly quantitative correlation between signal and local concentration of contrast agent, MRI is tomographic and can acquire images with a high spatial resolution. Therefore, MRI, unlike nuclear imaging, can be used to study directly the abundance and tissue distribution of transplanted islets. 

The innate low sensitivity of MRI can be overcome with the use of contrast agents, such as superparamagnetic iron oxide nanoparticles. Superparamagnetic iron oxide nanoparticles have been extensively used as magnetic resonance reporters. Their basic structure includes an iron oxide core covered with a dextran coat [75] that can be functionalized with additional imaging, targeting, or therapeutic moieties. The presence of iron oxides in tissue is evidenced by a loss in signal intensity on T2-weighted and T2*-weighted MR images or, in technical terms, by a shortening of the T2 relaxation time of surrounding water protons. 

**Figure 1:**
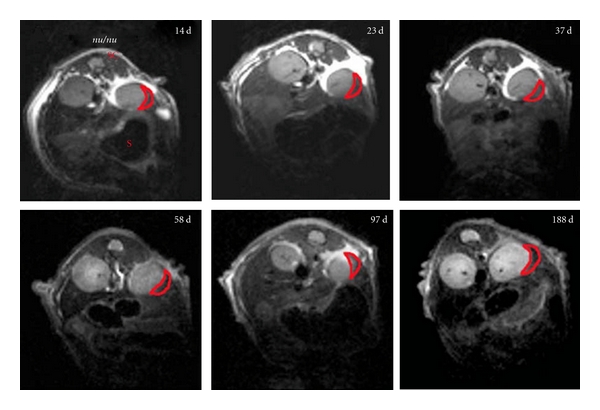
Transverse T2-weighted magnetic resonance images of transplanted labeled and nonlabeled human islets 14, 23, 37, 58, 97, and 188 d after transplantation under the kidney capsule in nude (nu/nu) mice. The dark area in the left kidney represents a labeled graft (red outline). No darkening was reported for the right kidney with unlabeled graft. S: stomach; SC: spinal cord, reproduced with permission from Nature Publishing Group [67].

Several groups focused on superparamagnetic iron oxides [67, 76, 77] for the labeling of isolated pancreatic islets before-transplant, followed by their noninvasive monitoring using MRI. In the study performed in our laboratory, isolated human pancreatic islets were labeled with superparamagnetic iron oxide magnetic nanoparticles (MNs) modified with a near-infrared fluorescent dye (MN-NIRF) and transplanted under the kidney capsule in immunocompromised mice. MN-NIRF-labeled human pancreatic islets were visualized for up to 188 days after transplantation in this model demonstrating graft stability and persistence of the label (Figure 1). The labeled islets were viable and functional, since the graft could restore normoglycemia in diabetic mice [67].

In more recent experiments, we also demonstrated the applicability of the approach to the intrahepatic transplantation model. For islet labeling we utilized an FDA-approved commercially available iron oxide agent (ferumoxides), which is routinely used in the clinic for liver imaging. Similar to MN-NIRF, it consists of superparamagnetic iron oxide covered with a dextran coat. Human islets labeled with ferumoxides and transplanted into the liver appeared as distinct hypointense foci representing single islets and/or islet clusters. The persistence of the graft in immunocompromised animals was demonstrated for the entire observation period of two weeks [78].

The applicability of MRI for the visualization of transplanted islets, following their labeling with a contrast agent, was also demonstrated using paramagnetic beads [79], and a paramagnetic contrast agent, GdHPDO3A [77]. In another study, the strong relaxation effect of superparamagnetic iron oxides allowed the detection of transplanted islets at a lower magnetic field strength (1.5 T), equivalent to the ones used in hospitals today, advancing the method to a more clinically relevant stage [80].

Visualizing transplanted islet in large animal models using MRI serves as an important step before clinical application. The first study in large animals was reported in 2007 [81]. Human islets labeled with immunoprotective iron oxide-loaded magnetic capsules were detected with real-time MRI [81]. Recently, our group reported the in vivo imaging of autologous islet grafts in the liver and under the kidney capsule in nonhuman primates. The renal subcapsular islet grafts were easily detectable on T2*-weighted MR images as a pocket of signal loss disrupting the contour of the kidney at the transplantation site. Islets transplanted in the liver appeared as distinct signal voids dispersed throughout the liver parenchyma. This study established a method for the noninvasive, longitudinal detection of pancreatic islets transplanted into non-human primates using a low-field clinical MRI system [25]. 

The first imaging study applied in humans with superparamagnetic iron oxide nanoparticles was carried out in 2008 [82]. Islets were labeled with superparamagnetic iron oxide particles (SPIO, 280 microg/mL) and transplanted into patients with T1DM. All patients could stop insulin after transplantation. Three out of four patients had normal intensity on pretransplant images, and iron-loaded islets could be identified after transplantation as hypointense spots within the liver. However, this clinical study did not show any correlation between the number of labeled transplanted islets and the number of hypointense spots on MR images. In addition, the number of spots varied significantly over the course of the study making it impossible to make any conclusions regarding graft outcome. In spite of these shortcomings the study demonstrated that islet labeling is safe and islet function is not affected by labeling. 

## 4. Imaging of Islet Grafts Rejection

### 4.1. Fluorescence Optical Imaging

The earliest report utilizing fluorescence optical imaging for the monitoring of immunological effects associated with islet transplantation relied on genetic engineering to create transgenic mice that express proinsulin II fused with the live-cell fluorescent reporter protein, Timer [66]. Since Timer protein changes its emission wavelength in the first 24 hr after synthesis, it can be used to monitor the time course of insulin synthesis. Islets expressing this construct were transplanted into recipient transgenic mice in which T lymphocytes were fluorescently labeled with a fluorochrome different from Timer. The animals were monitored through a body window device to derive complementary information about insulin synthesis and alloimmunity triggered by the engrafted islets. The value of this method lies in the fact that, for the first time, it allows the direct analysis not simply of islet abundance but also of islet function in the context of immune rejection. 

Recently, Fan et al. have developed a new reporter mouse model, which has its T-cell expressing distinct “color-coded” proteins enabling in vivo detection of different T-cell subsets. With these tools, the authors found notable differences in the T cell response in islet grafts recipients receiving tolerance-inducing treatment compared to control group. These studies established real-time cell tracking tool to probe the islet grafts immunologic rejection at cellular level [83].

### 4.2. Bioluminescence Imaging

A comprehensive investigation into the relative effects of immune rejection on viable islet mass was obtained by the transplantation of islets obtained from a transgenic mouse strain, which constitutively expresses firefly luciferase, underneath the renal capsule or into the liver of syngeneic or allogeneic streptozotocin-induced diabetic recipients. Whereas, in isografts, following an almost 50% decrease in signal intensity in the first 14 days after transplantation, there was an overall long-term stability of luminescence intensity signals, in allografts, graft bioluminescent intensity progressively decreased several days before the permanent recurrence of rejection-induced hyperglycemia [65]. In nontransgenic syngeneic recipients transplanted with transgenic islets, high levels of bioluminescence over the abdominal region of the liver were detected within 24 hr after [64]. Monitoring bioluminescence signal from the abdomen of the recipient for more than 90 days revealed a decline over time. With the caveat that bioluminescence signal can be influenced by a variety of factors, such as serum glucose levels, mouse positioning, and surgical and motion artifacts, and so forth, this work suggests that in the absence of immune rejection islet grafts survival can be extended significantly. Even though this conclusion is not surprising, the described method lays the groundwork for future studies in which various immunosuppression strategies can be tested for their potential to more closely emulate a syngeneic immune context.

### 4.3. Magnetic Resonance Imaging

**Figure 2:**
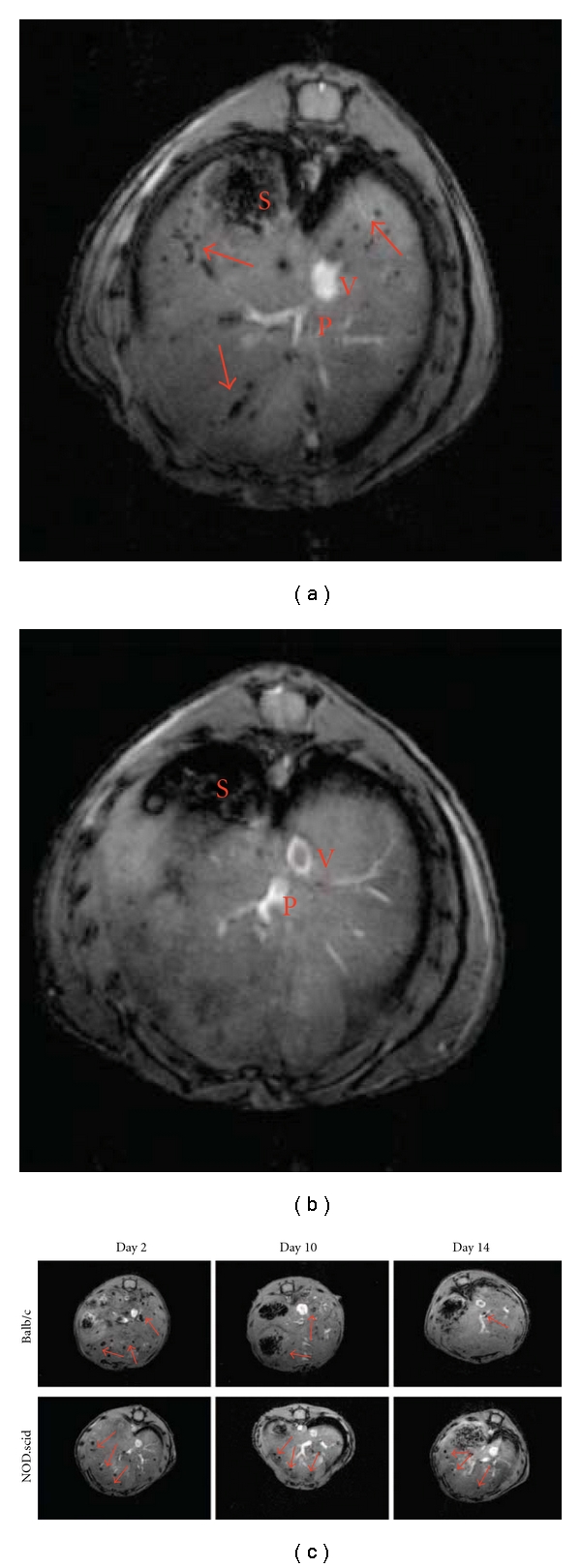
In vivo imaging of intrahepatically transplanted human islets. (a) Representative images of NOD.scid mice with transplanted islets. On in vivo images, Feridex-labeled islets appeared as signal voids scattered throughout the liver. (b) Nonlabeled islets were not detectable using the same imaging parameters. (c) In vivo time course imaging of immune rejection in immunocompetent (upper row) and immunocompromised (lower row) animals. Representative images are shown from days 2, 10, and 14 after transplantation. Note that the signal voids (arrows) representing labeled islets/islet clusters tend to disappear faster in Balb/c mice, reproduced with permission from American Diabetes Association [78].

Our group conducted similar studies to the ones described above in which we employed a pre-clinical model of islet transplantation at the hepatic site where islets were monitored by MRI. Immunocompetent Balb/c mice exhibited a significantly higher rate of islet loss as seen on MR images, compared to immunocompromised animals. Islet loss in the immunocompetent model was especially pronounced on day 10 after transplantation and ultimately resulted in a 20% difference in relative islet number by 14 days after transplantation (Figure 2) [78]. This report established a quantitative framework to describe the rate of islet loss in an immunocompetent context. Because of the direct comparison between the immunocompetent and immunocompromised models, this quantitative, noninvasive, and time-course sensitive imaging method allowed us to isolate out the relative contribution of allorejection to the overall decrease in transplanted islet mass in the early posttransplant period from among a multitude of factors, such as mechanical stress and vascular disruption [84].

## 5. Imaging of Novel Islet Transplant Sites

### 5.1. Bioluminescence Imaging

The clinically relevant site of islet transplantation is the liver. In research models, islets are also routinely transplanted underneath the renal capsule. Still, because of the suboptimal performance of islet grafts at these two sites, there is an ongoing effort to identify and test novel transplantation sites that provide a more suitable vascular environment for the islets, as well as a more immunoprotected physiological niche. The epididymal fat pad has emerged as an alternative transplant site, characterized by similar outcomes to intraportal transplantation. Mouse islets implanted into the intra-abdominal epididymal fat pad restored normoglycemia in STZ-treated recipients [85]. As few as 50 islets mediated similar levels of glucose tolerance when transplanted in the fat pad and the liver. When transgenic luciferase-positive islets were transplanted, bioluminescence imaging showed stability of the grafts for over 5 months. This was further supported by histological examination of the grafts showing healthy, well-granulated insulin-containing cells surrounded by healthy adipocytes [85]. These experiments suggest that the fat pad may be an alternative site of islet transplantation that is characterized by outcomes at least equivalent to the intraportal route.

### 5.2. Laser Scanning Microscopy (LSM)

A very interesting study explored the anterior chamber of the eye as a site of islet transplantation [86]. The anterior chamber of the eye is a suitable transplantation site because it provides an immune-privileged environment and because the high amount of autonomic nerves and blood vessels found in the iris enables fast engraftment. Islets were isolated from transgenic mice expressing enhanced GFP under the control of the rat insulin-1 promoter (RIP-GFP) and used for transplantation. Simultaneous two-photon LSM (TPLSM) of beta cells (GFP signal) and the vascular network (intravenous Texas Red-conjugated 70-kDa dextran) revealed that, by day 3 after transplantation, islets attached to the iris and began to recruit blood vessels from the iris. By day 14, blood vessels formed a microvascular network throughout the islet grafts [86, 87]. 

Furthermore, the authors monitored changes in cytoplasmic free Ca^2+^ concentration of the transplanted islets, as a direct indicator of islet function. Islets were loaded with the Ca^2+^ indicators Fluo-4 and Fura-Red via perfusion of the anterior chamber of the eye and imaged using LSM. Changes in cytoplasmic free Ca^2+^ concentration were successfully measured following stimulation of beta-cells activity with glibenclamide [86, 87]. 

Finally, the authors used LSM to noninvasively image beta cell death in islets transplanted into the anterior chamber of the eye. RIP-GFP islets were implanted into the anterior chamber of the eye and, after complete engraftment and vascularization, monitored after intravenous administration of the fluorescently labeled apoptotic marker annexin V. These experiments revealed a very low incidence of cell death in islets transplanted in this site most likely due to its immune-privileged surroundings [86, 87].

These combined studies illustrate the potential of noninvasive imaging for the collection of comprehensive information about transplanted islet biology and function on a systemic level. Islet engraftment, vascularization, function, and cell death were for the first time examined within a coherent experimental framework, providing unique knowledge on the subject of islet transplantation. 

Most recently, the same group reported that they used intravital multiphoton microscopy to monitor transplanted islets in the anterior chamber of the mouse eye. This technique allowed for studies at the single-cell resolution and enabled longitudinal, noninvasive imaging of immune response within target tissues during islet allorejection [88].

### 5.3. Nuclear Imaging

Lu et al. [70] implanted islets adenovirally transduced with sr39tk into the axillary cavity of recipient animals. The reason the authors selected this transplantation site is because untrapped PET probe is eliminated through the gut and kidney and can create spillover background signals in the pancreas, kidney, and liver regions of small animals until it is excreted. Therefore, to avoid background signals, the authors initially tested whether sr39tk-expressing islets could be imaged after implantation into the axillary cavity, which is far from the probe elimination pathway. In this model, there was a direct relationship between microPET signal and implanted islet mass. In longitudinal studies, signal decreased by approximately one-half during the first few weeks after transplantation, followed by stabilization over 90 days, suggesting significant islet cell death in the immediate posttransplant period. Ex vivo analysis demonstrated that sr39tk-expressing islets transplanted into the axillary cavity had normal morphology, were healthy and positive for insulin, glucagon, and somatostatin, and had no evidence of inflammation [70].

## 6. Combination of Imaging and Therapeutic Interventions

Therapeutic interventions for the enhancement of islet grafts performance can include immunomodulatory treatments of the graft or of the host to minimize allorejection or autoimmunity, methods to expedite graft revascularization, and approaches aimed at optimizing islet function or survival. The potential of noninvasive imaging to evaluate these treatments is an important part of their success. In addition, novel technologies may provide tools combining imaging and delivery of experimental therapies that aim to extend the lifespan and functionality of islet grafts.

### 6.1. Bioluminescence Imaging

In one of the earliest reports using BLI to evaluate a novel therapeutic strategy for transplanted islet immunoprotection, canine pancreatic islets were encapsulated into biocompatible alginate beads and transplanted into the dorsal-cervical fat pad of a nuclear factor-kappa beta (NF-*κ*B) luciferase transgenic mouse model [89]. Mice were imaged by BLI for up to 45 days after transplantation to evaluate the long-term inflammatory response provoked by the transplantation. The results suggested that the trauma of surgery was a more significant inflammatory trigger than host immune responses to the capsules and that capsule size, rather than composition, correlated with an increase in inflammatory activity in this model. This study is valuable because it establishes the feasibility of a method that can be used to assess the extent to which various immunomodulatory strategies provoke an inflammatory response in the context of islet transplantation.

A more recent investigation attempted to determine whether early detection of rejection by BLI could aid in the timing of antilymphocyte serum (ALS) treatment for prolonging islet grafts survival [90]. Transgenic islets expressing the firefly luciferase were transplanted under the kidney capsule of streptozotocin-induced diabetic allogeneic immunocompetent mice. The animals received anti-lymphocyte therapy whose effect on islet survival was monitored by BLI. Imaging was proven useful in designing an optimal timing of therapy administration resulting in a close to 60% reduction in grafts loss from rejection. Interestingly, the same success could not be achieved using blood glucose levels as a guide for the timing of therapy.

### 6.2. Nuclear Imaging

Nuclear imaging has been used to determine the feasibility of PET reporter gene (PRG) and PET reporter probe (PRP) technology for tracking islet grafts survival and quantifying the expression of potential therapeutic genes [71]. The authors generated a dual gene—expressing recombinant adenovirus rAD-vIL10-ITK, which coexpresses viral interleukin-10 (vIL-10) and HSV1-sr39tk. vIL-10 was chosen because it protects transplanted islets from immunological attack by regulating autoimmune activity. Islets from nondiabetic NOD mice were infected with rAD-vIL10-ITK or rAD-ITK, transplanted under the right kidney capsule of diabetic NOD mice, injected with [18F] FHBG, and scanned by microPET. PET signals in mice transplanted with islets infected with rAD-ITK (rAD-ITK mice) were decreased at 3 d and had almost reached basal values at 14 d after transplantation. Imaging revealed a relative extension of islet grafts survival in the presence of IL-10 treatment [71], illustrating the potential utility of anti-inflammatory therapy in islet transplantation.

### 6.3. Magnetic Resonance Imaging

Magnetic resonance imaging also has been explored for the pre-clinical assessment of therapeutic interventions in the context of islet transplantation. The first study was done in a large animal model (swine) and described the application of immunoprotective magnetocapsules containing ferumoxides, as carriers of pancreatic islets [81]. Islet-containing magnetocapsules were infused and engrafted into the liver. MRI could monitor this process in real time because of the incorporation of ferumoxides into the capsules. 

A similar multifunctional rationale aimed at exploring the possibility for concurrently imparting imaging and therapeutic capabilities to transplanted pancreatic islets has also been behind some of our most recent work [92]. Taking advantage of the propensity of pancreatic islets to avidly take up dextran-coated superparamagnetic iron oxide nanoparticles, we designed a probe that consisted of a dextran-coated iron oxide core, conjugated to small interfering RNA (siRNA). Considering the potential of siRNA as a novel class of small molecule drugs capable of selectively silencing the expression of essentially any gene of choice with single nucleotide specificity, we speculated that we could explore the mechanism of RNA interference in the context of islet transplantation. As proof of concept, we showed that siRNA tagged to magnetic nanoparticles could accumulate in pancreatic islets in quantities sufficient for detection by MRI in vitro and for silencing target genes (green fluorescent protein [GFP] was used as a model gene) [92]. 

**Figure 3:**
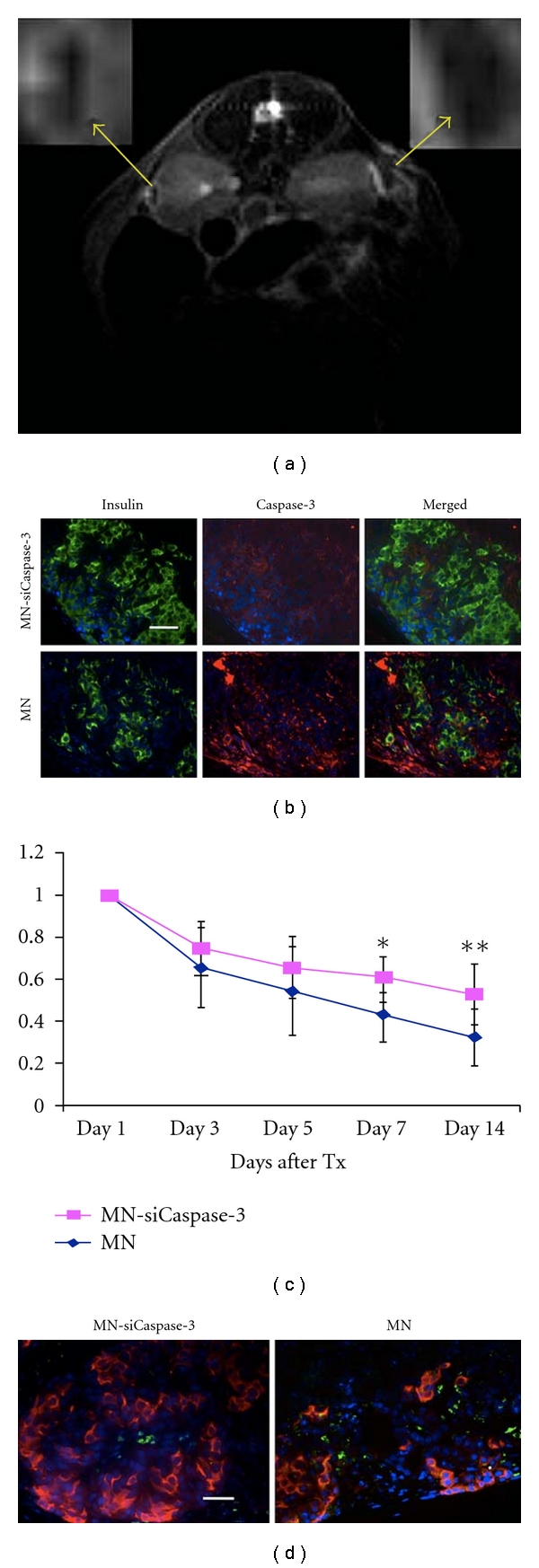
(a) Representative in vivo MRI of islet transplantation showing MN-siCaspase-3-treated islets implanted under the left kidney (inset) and parental MN-treated islets implanted under the right kidney. The dark area outlined under both kidney capsules represents the labeled grafts (day 3 shown). (b) Semiquantitative assessment of the relative changes in graft volumes revealed protective effect in MN-siCaspase-3-labeled grafts (*day 7, *P* < 0.05; **day 14, *P* < 0.05). (c) Fluorescence microscopy revealed higher expression of insulin and lower expression of caspase-3 in MN-siCaspase-3-treated grafts compared with MN-labeled islet grafts on the 14th day after-transplantation (Tx) (green, insulin; red, cleaved caspase-3; blue, DAPI nuclear stain) (magnification bar = 50 mm). (d): TUNEL assay on insulin-stained sections confirmed lower apoptotic rate and higher insulin expression in islets treated with MN-siCaspase-3 compared with islets treated with MN on the 14th day post-Tx (red, insulin; green, TUNEL; blue, DAPI nuclear stain) (magnification bar = 50 *μ*m), reproduced with permission from American Diabetes Association [91].

A more recent study by our group used a dual-purpose therapy/imaging nanoparticle probe to target the apoptotic-related gene caspase-3. We demonstrated that our “two-in-one” MN-siCaspase-3 imaging probe could silence the apoptotic-related gene, providing significant protection to the grafts from early loss after transplantation, and at the same time served as an MRI label to assess the in vivo post-transplant fate of the grafts noninvasively (Figure 3) [24, 91]. The results of our study are in line with those of another recently published study showing that the use of fluorinated alginate microcapsules increased the insulin secretion rate of human islets and at the same time allowed detection by MRI and CT imaging [94].

In the context of islet transplantation, these studies are valuable because they lay the groundwork for future applications, in which genes implicated in islet grafts loss (immunological or nonimmunological) can be similarly targeted in order to improve graft outcome. Furthermore, the inherent imaging capabilities of the approach permit the noninvasive tracking of therapeutic moiety conjugated to the contrast agent and its relationship to graft fate.

## 7. Imaging of Metabolic Effects on Islet Grafts

One factor normally present during clinical transplantation and influencing transplanted islet fate is glucotoxicity caused by chronic hyperglycemia. To assess its input to the graft outcome, our group labeled human pancreatic islets with iron oxides and transplanted them into hyperglycemic and normoglycemic animals [95]. Noninvasive MRI monitored the fate of the grafts in the two groups. We found that in diabetic animals there was a significantly higher rate of islet death than in the normoglycemic counterparts. The half-life of an islet in the diabetic group was 4.8 ± 1.1 days compared with 12.6 ± 2.9 days for control nondiabetic mice (*P* < 0.05). This is the first in vivo study that confirms previous in vitro reports demonstrating that severe hyperglycemia impairs graft function and that successful islet transplantation depends on the degree of hyperglycemia in the recipient [96–103].

## 8. Imaging of Stem-Cell-Derived Insulin-Producing Cells

Pluripotent stem cells have the potential to differentiate into specialized cells of all three primary germ layers. Embryonic stem (ES) cells and the newly developed induced pluripotent stem (iPS) cells are an ideal source for generating insulin-producing cells (IPCs) that could be used to treat diabetes. 

Most recently, BLI was used for monitoring ES cell survival and differentiation into insulin-producing cells in a diabetic animal model. This group generated a double transgenic mouse ES cell line ectopically expressing Pdx1-aequorea coerulescens green fluorescent protein (AcGFP) fusion protein, and rat insulin promoter- (RIP-) driven luciferase reporter [93, 104]. Real-time noninvasive BLI was used to monitor cell fate and function after transplantation. They speculated that, in vivo, pancreatic endoderm-like cells (PELCs) migrate into the streptozotocin-damaged pancreas and differentiate into IPCs. The in vivo differentiation of double transgenic ES cells transplanted under the renal capsule or systemically infused cells could be imaged by BLI as early as day 3 and until day 35 after-transplantation [93].

## 9. Conclusions

Pancreatic islets, when transplanted, should be able to restore glucose homeostasis in the patient with diabetes. Nevertheless, the processes behind islet isolation, delivery, engraftment, and proper functioning in a new metabolic and immunological environment are complex and still poorly understood. Here, we have briefly discussed the important questions related to islet transplantation that could be tackled through in vivo molecular imaging. They bridge a range of topics, including graft longevity in the context of immune rejection and hyperglycemia, effects of autoimmunity and strategies to alleviate immune-mediated deterioration of graft viability and function, and the exploration of novel transplantation sites. 

Still, despite the significant value of these studies, they reflect only a small proportion of the issues that face the field of islet transplantation. Questions that remain unresolved or need to be studied in more detail span from the mechanics of islet engraftment and vascularization to the molecular aspects allowing islets to adapt to a new immunological, physiologic, and metabolic niche distinct from the pancreas, the importance of factors, such as islet innervation, ambient metabolite, ion, or signaling molecule concentrations for proper islet function, and so forth. Furthermore, having in mind the limited supply of donor tissue, another crucial subject to investigate would involve the potential of transplanting nonislet tissue, such as stem cells or cell types that can be induced to differentiate or transdifferentiate into functional islet tissue. 

Since molecular imaging has the advantage of delivering quantitative temporal information in intact, authentic physiologic environments and on a systems' level, its potential for comprehensively addressing the issues of immediate interest to islet transplantation is apparent. Development of probes targeting biomarkers linked to beta-cell function and combining therapeutic and diagnostic (theragnostics) tools for improving islet transplantation should be encouraged. With the first feasibility steps having been already made, the near future should see rapid progress.
